# Unlearning clubs: creating environments of cultural safety, anti-racism, and trustworthiness in population and public health

**DOI:** 10.1186/s12889-025-22034-6

**Published:** 2025-03-24

**Authors:** Jorden Hendry, Giuliana Del Guercio, Danièle Behn Smith, Amber Louie, Bonnie Henry, Kate Jongbloed

**Affiliations:** 1 University of British Columbia, Vancouver, British Columbia Canada; 2 BC Office of the Provincial Health Officer, Victoria, British Columbia Canada; 3 University of Victoria, Victoria, British Columbia Canada; 4 British Columbia Centre for Disease Control, Vancouver, Canada

**Keywords:** Anti-racism, Public health, Education, Indigenous health

## Abstract

**Background:**

Unlearning is a necessary practice for disrupting the deeply ingrained colonial narratives and racialized assumptions that sustain racism in Canada. We created and implemented an “Unlearning Club” in British Columbia’s Office of the Provincial Health Officer (OPHO). It is a structured and on-going space where public health professionals and trainees critically engage with Indigenous rights, anti-racism, anti-white supremacy, and cultural safety.

**Methods:**

The Unlearning Club and the reflective research presented in this paper draw on the frameworks of Dr. Camara Jones and Jody Wilson-Raybould to guide three key processes: naming racism and white supremacy (LEARN), asking, how are they operating here (UNDERSTAND), and organizing and strategizing to act (ACT). To document teachings and reflections, we used two approaches. Participants completed a structured rapid reflection tool (*n* = 67) after each session to capture experiences and insights. Second, Unlearning Club hosts engaged in a relational reflection process, allowing for deeper discussion on lessons learned and future directions.

**Pragmatic reflections:**

(1) *Spotlighting Land Acknowledgement*: starting each session with a more in-depth land acknowledgments allowed participants to ground their unlearning in the place-based inherent rights of Indigenous Peoples. (2) *Relational Accountability*: Unlearning Club nurtured solidarity and accountability among colleagues, giving tools needed to move into action. (3) *Sustained Unlearning*: The process of unlearning led to profound shifts in perspective, as individuals were unable to “unsee” pervasive ways in which white supremacy operates, moving individuals beyond feeling paralyzed by injustice. (4) *Collective Responsibility*: The cohort model allowed relationship-building and fostered shared commitment to addressing racism and white supremacy, creating a stronger group-wide sense of purpose and accountability. (5) *Counters Resistance*: Eagerness and gratitude was widespread among participants; individuals were willing to invest the time and effort necessary to engage deeply in the unlearning process. (6) *Moving to Action*: Participants highlighted the effectiveness of the “Learn, Understand, Act” framework in preventing stagnation. This structured approach offered clear pathways to translate new insights into tangible actions.

**Conclusion:**

The Unlearning Club has emerged as a pivotal strategy for fostering environments of cultural safety, anti-racism, and trustworthiness within our public health office. Sustained engagement in anti-racist education, supported by strong leadership and an inclusive structure, can significantly reduce resistance to anti-racism initiatives.

## Introduction

### Introducing ourselves

We are a team of Indigenous and non-Indigenous public health leaders working in what is known colonially as “British Columbia” (BC). We came together with the overarching purpose of advancing the rights and health of Indigenous Peoples by dismantling racism and white supremacy within public health systems. Ms. Jorden Hendry (Tsimshian, Lax Kw’alaams/settler) is a doctoral student at University of British Columbia. Ms. Giuliana Del Guercio (Chicana/Mestiza) is a former OPHO U&U Research Assistant. Ms. Amber Louie (settler/Ktunaxa Nation, Yaqan Nuʔkiy community) joined our team as an MPH student at University of Victoria. Dr. Danièle Behn Smith (Eh Cho Dene Fort Nelson First Nation & Métis Red River Valley) serves as Deputy Provincial Health Officer, Indigenous Health in BC’s Office of the Provincial Health Officer. Dr. Bonnie Henry (White settler) serves as the Provincial Health Officer in BC’s Office of the Provincial Health Officer. Dr. Kate Jongbloed (White occupier) served as a post-doctoral fellow in BC Office of the Provincial Health Officer and an Associate with Qoqoq Consulting Ltd. The aim of this paper is to share how we developed and implemented “Unlearning Clubs” as part of our work to create environments of cultural safety, anti-racism, and trustworthiness.

### Why unlearning?

We use the term *unlearning* to shine a light on the insidious but ubiquitous socialization of Indigenous-racist attitudes and behaviors. We have all ‘learned’ to uphold Indigenous-specific racism simply by being raised in ‘Canada.’ Lavallee and Harding have argued that Indigenous-specific racism is coached into Canadian health systems via a “hidden curriculum” that upholds negative stereotypes [1]. Others have used the term “colonial scripts” to define this hidden curriculum that is pervasive within mainstream Canadian education [2]. “Colonial scripts” are the stories, narratives, and statements that frame Indigeneity as inferior while simultaneous constructing white settler identity as superior effectively naturalizing settler colonial power [2]. Unlearning Club shifts the focus away from learning about Indigenous Peoples, histories, or cultures and instead challenges settlers to critically examine their own roles in maintaining settler colonialism. In many reconciliation and cultural safety initiatives, ‘learning’ is often framed in ways that position Indigenous peoples as both the problem and the solution—without triggering necessary self-reflection or self-implication. Unlearning is necessary because it actively disrupts the deeply ingrained colonial narratives and racialized assumptions that shape everyday attitudes, policies, and systems [2]. Only through unlearning can genuine re-learning take place—one that is rooted in accountability, critical self-reflection, and a commitment to dismantling settler colonial systems rather than merely adapting to them.

Most individuals currently working in Canadian public health systems have not received formal training in Indigenous-specific anti-racism throughout their academic journey. From primary school through post-secondary education, curricula have largely failed to address history of settler colonialism, systemic racism, and Indigenous rights. Unlearning Club was designed to fill this gap by creating a structured, sustained space for public health professionals to critically engage with Indigenous-specific anti-racism, cultural safety, and the dismantling of white supremacy. The primary goal is to foster ongoing learning, self-reflection, and action, equipping participants with the knowledge and skills necessary to challenge and change the status quo in public health systems. In our context, “unlearning” refers to how the materials and perspectives included within our syllabus serve as a counter narrative. It reflects our acknowledgement that we and our colleagues have been taught the “hidden curriculum” of colonial narrative and through exposure to viewpoints from Indigenous, Black, and Peoples of Color we can begin a process of dismantling that scaffolding and building anew.

### Racism as a public health crisis

Racism as a public health crisis is profoundly impacting the health and wellbeing of individuals and communities [3–7]. The social construction of race was created to protect racial hierarchies that positioned White people at the top and Indigenous, Black, and Peoples of Color at the bottom [8, 9]. Dr. Camara Jones defines racism as “system of structuring opportunity and assigning value based on the social interpretation of how one looks, that unfairly disadvantages some individuals and communities, and unfairly advantages other individuals and communities” [10]. In Canada & British Columbia, Indigenous-specific racism in health is widespread and reflects the White supremist origins of Canadian governments [11–13]. In the 2020 *In Plain Sight: Addressing Indigenous-Specific Racism and Discrimination in BC Health Care* report, 84% of 2,780 Indigenous survey participants experienced discrimination when receiving health care [14–16]. This report aligns with longstanding calls to address Indigenous-specific racism, including the *Truth and Reconciliation Commission of Canada’s Calls to Action* [15], particularly Calls 18–24, which focus on closing health disparities and ensuring cultural safety. Similarly, the *Final Report of the National Inquiry into Missing and Murdered Indigenous Women*, *Girls*, *and 2SLGBTQQIA +* [16] calls for immediate systemic changes to eliminate racism in health (Calls for Justice 3.1–3.7). In British Columbia, the adoption of the *Cultural Safety and Humility Standard* [17] by health authorities further underscores the need for actionable commitments to anti-racism and reconciliation in health systems. Collectively, these reports provide a clear and urgent directive on eliminating Indigenous-specific racism and advancing reconciliation. Obligations to uphold inherent Indigenous rights, anti-racist approaches, and truth and reconciliation is articulated in provincial, federal, and international laws [18–21]. As public health professionals, we all have obligations to reject the racist status quo by identifying, describing, and dismantling the ways in which we continue to facilitate racism though policy and practice [22, 23]. Unlearning inherited White supremist structures requires a deliberate shift in our understanding and is integral to anti-racism efforts as described by Ibram X. Kendi, “To be antiracist is a radical choice in the face of history, requiring a radical reorientation of our consciousness.” [24].

### Daylighting three intersecting systems of oppression

An essential step for dismantling Indigenous-specific racism is to make visible three intersecting systems of oppression: white supremacy, settler-colonialism, and racism. The current colonial health system in Canada has been built and led by White people with Eurocentric knowledge; because of this, deeply embedded ideologies of white Supremacy continue to confer unearned advantage on a narrow segment of the population, while others experience active harm [13, 25, 26]. Consequently, the prevailing ‘status quo’ within Canada’s public health systems is characterized by the legacy of colonization and a system steeped in White supremacist ideologies; unless we are consistently and coherently working to be *anti-racist*, we risk upholding racism that is normalized in the system [12, 27–29]. Understanding these three intersecting systems of oppression shapes how we understand public health issues. In this article we provide an approach to anti-racist training, where we can collectively offer new solutions to public health issues that strive not only to dismantle deep-rooted racial biases but also to move towards eliminating the negative health effects from racism [7]. The Unlearning Club shares and discusses resources from Indigenous, Black, and People of Color to build our capacity to critically understand and dismantle the influence of these systems of oppression in public health.

### Research context & unlearning club structure

This research is set within the BC Office of the Provincial Health Officer (OPHO). As the senior public health official for the westernmost Canadian province of British Columbia (BC), the Provincial Health Officer is responsible for monitoring the health of the population of BC and providing independent advice to ministers and public officials on public health issues [30]. In 2021, we launched the “Unlearning & Undoing white supremacy and Indigenous-Specific Racism Project” within the OPHO to illuminate which structures, policies, practices, norms, and values in our office uphold systemic white supremacy and racism. Key to this process has been reflecting on our own relationship(s) to settler colonialism and white supremacy as individuals and as a collective.

We recognize the importance of *unlearning* as a means to strengthen *undoing*. As a result, we created an OPHO Unlearning Club: a monthly structured space that provided dialogue and self-directed unlearning related to Indigenous rights, anti-racism, anti-white supremacy and cultural safety. A closed cohort of OPHO staff and leaders were organized into racial affinity groups of 6–8 members to learn from and discuss resources created by Indigenous, Black, and People of Color scholars and experts. Wise practices for this type of unlearning work recommend that white, Indigenous, and racialized people meet separately [31, 32]. While this approach may seem counter-intuitive, the literature indicates that due to differing experiences in relation to white supremacy and racism, meeting separately helps to mitigate harm that can be caused by white fragility and support critical reflection on settler identities [33, 34]. Affinity groups were used to allow for a more honest and open environment, and to mitigate harm to racialized peoples. White people processing their emotions about racism often unintentionally center themselves in discussions, placing the burden on racialized participants to witness, comfort, or educate them, which affinity groups help prevent. This structure also allows racialized participants can focus on creating community and strategies for navigating white supremacy rather than engaging in white people’s learning process.

We developed 17 two-hour sessions that are organized into three modules, following Dr. Camara Jones’ Science & Practice of Anti-Racism [10]. The length of the Unlearning Club reflects the necessarily slow and sustained effort required to shift entrenched norms and practices of white supremacy. Cultural change does not happen overnight, and as Jody Wilson-Raybould emphasizes, unlearning must be a “conscious, coherent, consistent” [12] process rather than a one-time intervention. Interspersed throughout the Unlearning Club are months that we refer to as “breaths of fresh air” which shift focus from racism to Indigenous ways of knowing and perspectives on health and wellness.

## Methods

### Research & practice frameworks

**Fig. 1:**
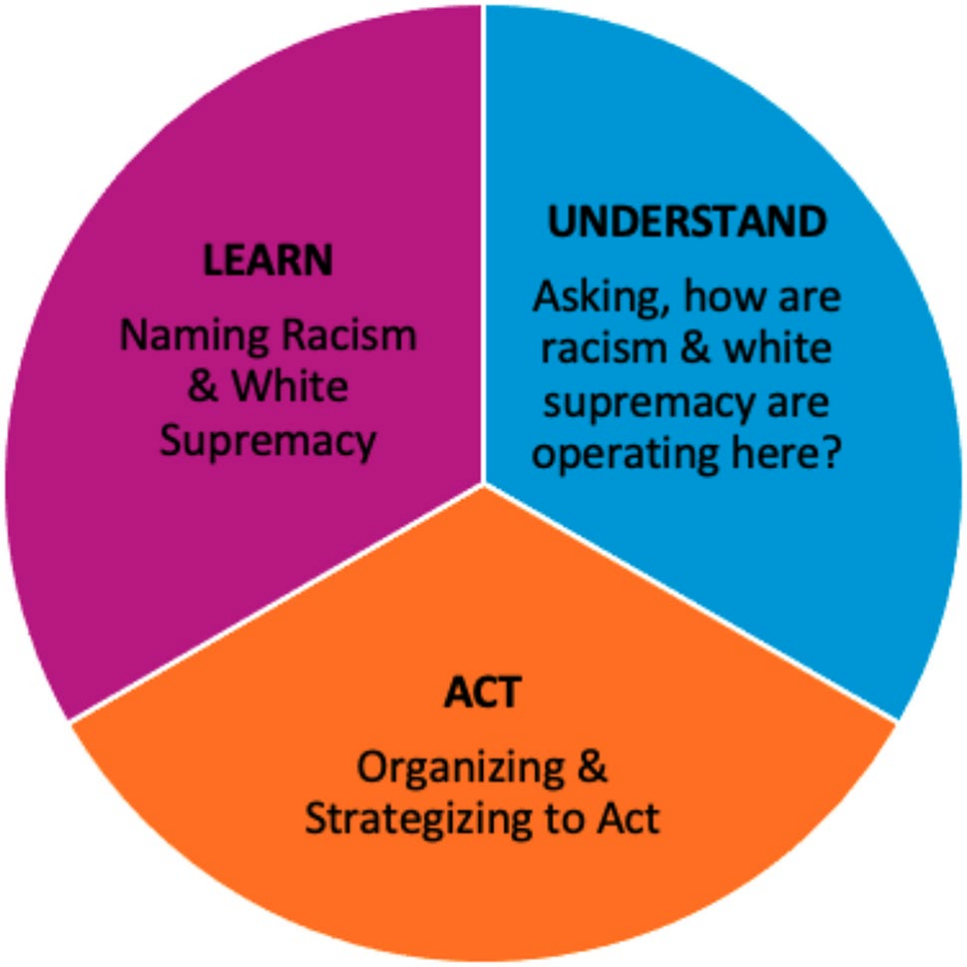
Unlearning club frameworks: Dr. Camara Jones’ “Science and Practice of Anti-Racism” & Puglaas Jody Wilson Raybould’s “True Reconciliation”

The Unlearning Club and the reflective research presented in this paper utilizes two closely related frameworks, firstly, former American Public Health Association president Dr. Camara Jones’ “Science and Practice of Anti-Racism” framework: (1) naming racism; (2) asking, how is it operating here?; and (3) organizing and strategizing to act [10]. Secondly, Kwakwakaʼwakw leader Puglaas Jody Wilson Raybould’s (JWR) “True Reconciliation” framework: (1) learn; (2) understand; and (3) act [10, 12]. While Dr. Camara Jones’ framework provided the foundation for structuring our modules and guided our reflections throughout the research, JWR’s *True Reconciliation* framework came to us during Unlearning Club, where we read “True Reconciliation: How to Be a Force for Change” [12] in month 10. Its alignment with our existing approach reinforced the importance of integrating a BC First Nations perspective; however, because it was introduced later in our process, it did not explicitly shape our initial findings. Instead, it has become a critical framework for informing our ongoing reflections and future applications of this work. Figure 1 represents the frameworks in a circle, to show that we move through them cyclically rather than in a linear way from start to finish. We must first name racism and understand it’s structural roots before we can move to action, to ensure it does not get repeated.

### Participants

Reflections by two types of Unlearning Club participants are presented in this paper:

#### OPHO unlearning club members

Staff and leadership (from all teams and roles) at the Office of the Provincial Health Officer actively employed in Spring/Summer 2022 were invited, but not required, to participate in the Unlearning Club. Members were permitted to undertake Unlearning Club prep work and sessions during work time. We shared information about the Unlearning Club at an office-wide meeting and hosted an open house for interested participants. We followed up with an email and asked those who were interested to reply. The sessions were held online. In total, 25 members participated over the course of the 17-months and were eligible, but not required, to be part of the research component. In some groups, members were learning alongside their supervisors; however, the senior leadership met separately. While this may have impacted openness, it was appropriate given that we aimed to strengthen our collective knowledge and skills (rather than individual knowledge and skills). All participants were split into closed cohorts, 3 groups were racial affinity groups (2 white, 1 racialized), and 1 leadership group.

Given that participation was voluntary, it is possible that individuals already engaged or interested in anti-racism were more likely to join, which may influence the generalizability of our findings. However, with over 70% of office staff participating, this high level of engagement suggests a strong sense of collective accountability and institutional commitment to unlearning and anti-racism work. All were welcome to participate, regardless of previous exposure to Indigenous-specific anti-racism and/or cultural safety training. The structure of Unlearning Club created space for diversity of previous experience in relation to the topic. We note, however, that San’yas Indigenous Cultural Safety Training is mandatory for all BC Ministry of Health employees. Thus, it may have functioned as a de-facto prerequisite.

#### Host-Members of the unlearning clubs

The hosts for the Unlearning Club sessions were existing members of the Unlearning and Undoing project team. Host-members play a multifaceted role, taking responsibility for prepping each session, including gathering resources, creating pre-reading homework, and disseminating it to other participants. During the sessions, the hosts presented a mini-module (e.g., brief presentation) of that month’s materials and facilitated discussions. While hosting was one responsibility, a significant portion of the hosts time was dedicated to being co-learners, fostering an inclusive and participatory learning environment. KJ & DBS hosted the OPHO Unlearning Club (JH & GDG hosted a modified version of the Unlearning Club for students at the School of Population and Public Health at the University of British Columbia, though not included part of this research).

### Data sources

#### Rapid reflections (longitudinal, during unlearning Club)

All OPHO members of the Unlearning Club were given the option of completing a 7-question anonymous online rapid reflection at the end of each session (Table 1). This approach has its foundations in the critical incident technique which has been used widely in a variety of settings [35–37]. In total, 67 were submitted. Some limitations of this data set must be noted. We aimed to provide 10 min at the end of each session for participants to complete the rapid reflection tool; however, in practice, this time was often given over to discussion about the session content. All participants were given the option to complete rapid reflections at every session. Participants did not need to respond to every question to submit their reflection. Given that responses were anonymous, we are unable to track individual consistency in participation. We did observe a decline in the number of responses over the 17-month period. This decrease aligns with the voluntary and repeated nature of participation, where engagement levels may naturally fluctuate over time.

**Table 1:** Rapid reflection on unlearning & undoing systemic white supremacy tool

Thinking about this month’s material & discussion:
1. What was/were the KEY LEARNING(S) for you this month?
2. Please reflect on anything about this month’s material or discussion that SURPRISED YOU.
3. Please reflect on any aspect of the material or discussion that made you UNCOMFORTABLE.
4. Please reflect on any aspect of this month’s material or discussion that felt AFFIRMING.
5. How does this month’s material or discussion relate to your personal or professional sphere of influence?
6. Please share any actions you took this month to undo systemic white supremacy or racism, or any actions you took to uphold the foundational commitments.
7. Is there anything else you would like to comment on?

#### Relational reflection (post completion of unlearning Clubs)

Four Unlearning Club host-members (JH, GDG, DBS, and KJ who are authors on this paper) undertook a process of relational reflection after the Unlearning Clubs came to an end to capture the unique insights as U&U team members being hosts, and active participants of the Unlearning Clubs. By actively engaging in reflective practices, we acknowledged and examined the intricate relationships among the researcher, participants, and the broader sociocultural context. These reflections, documented through collaborative notetaking, provided insights into the dynamic and evolving nature of the Unlearning Club. Documenting anti-racism and cultural safety work is not a straightforward process, relational reflections uncover the interplay between personal and contextual factors that truly capture the journey of the Unlearning Clubs. Relational reflections also provide a way to share insights from direct engagement with the participants not solely derived from predetermined research questions but also from the dynamic and reciprocal interactions between the researcher and participants.

### Analysis

The analysis was guided by Interpretive Description, our aim was to build on our existing knowledge and move toward identifying pragmatic learnings that support practice [38]. Given the reflective nature of the data, and being honest about its limitations, we chose to work towards a set of key pragmatic reflections rather than research findings. The analysis process began by gathering the Unlearning Club host-members to conduct relational reflections before reviewing participant data, allowing for preliminary identification of overarching reflections based on facilitation experiences. This was followed by an independent immersion in participant data, where host-members conducted in-depth analysis of the 67 Rapid Reflection responses, taking note of recurring themes, unexpected insights, and shifts in participants’ engagement over time. A second gathering with KJ and GDG was held, using a reflexive thematic analysis to group reflections into preliminary themes. Subsequently, JH independently reviewed the emerging pragmatic reflections, comparing them against the original participant responses to ensure that the themes accurately reflected the data. A third gathering was held with all Unlearning Club host-members to discuss and refine the emerging reflections, ensuring they were pragmatic and useful for guiding unlearning efforts. Finally, the process concluded with the finalizing and refining of the pragmatic reflections via email, checking for consistency in our interpretation of participant data and alignment in our relational reflections.

### Ethical considerations

Ethics for this study was approved by the University of Victoria’s Human Research Ethics Board. A letter of implied consent was distributed to all potential participants before the research began. Comprehensive details about the study were shared during Month 2 of the Unlearning Club, offering participants transparency about the research aims and procedures. We launched the first round of Rapid Reflection data collection in Month 3. Additionally, a reminder emphasizing the voluntary nature of participation was reiterated at the end of each session, just before providing the link to the online Rapid Reflection platform. Participation in the Rapid Reflection was anonymous. It was also reiterated that participation was in no way tied to work performance. These measures aimed to uphold ethical standards, prioritize participant welfare, and maintain a commitment to informed and voluntary participation throughout the study.

## Pragmatic reflections

Prior to implementing the Unlearning Club, OPHO setting was one of willingness and readiness to learn, but few had formal opportunities to build muscles in being anti-racist. We ensured the format of Unlearning Club was accessible for people at different stages of their learning journeys. We observed very high attendance in the Unlearning Club sessions over time. Most participants missed just one or two in the 17-month period, due to illness or holiday time. Most reported that despite their absence, they had completed their homework.

Participants found the format conducive to exploring complex topics related to racism alongside peers, allowing for a deeper and more nuanced comprehension of the issues at hand.

Six key reflections surfaced based on participant feedback and our experiences of hosting the Unlearning Clubs. We attribute the success and active participation of this club to these six key themes.

1. **Spotlighting the Land Acknowledgement: Unlearning Club was structured to strengthen our grounding in the inherent rights of the people on whose land it is taking place.**

Acknowledging and expressing gratitude to the original inhabitants of the territories where we live has become a common practice in our office [39, 40]. To strengthen the practice, each Unlearning Club participant took turns delivering a longer, more in-depth land acknowledgment of approximately 10 min, going beyond a typical one or two sentence statement. This pragmatic reflection emerged primarily from insights shared by the hosts, who engaged in relational reflections with one another. These reflections were informed by our own experiences facilitating Unlearning Clubs and being in relation with participants.

The monthly rotating land acknowledgment allowed us to ground our unlearning in the inherent rights of the peoples whose lands we are on, specifically recognizing and upholding the inherent rights to BC First Nations. This practice upholds many of the foundational obligations we have been given by Indigenous Peoples, such as United Nations Declaration on the Rights of Indigenous Peoples, Truth and Reconciliation Calls to Action, and others [15, 16, 18]. When holding the land acknowledgment responsibility, participants would spend significant time learning more deeply about the Nation and territories they occupy. The in-depth land acknowledgements would generally include slides, resources, and personal reflections of that person’s relationship to the land they inhabit. Participants also noted that they often undertook this learning and/or shared this learning with their families, so the impact of the in-depth land acknowledgements extended beyond the Unlearning Club. This responsibility rotated among participants each month and continued throughout the full 17-month duration of the Unlearning Club.

2. **Relational Accountability: The Unlearning Club format fostered trust among participants.**

The Unlearning Club fostered relational accountability among participants by creating a shared sense of responsibility and promoting accountability to a shared goal that extended beyond the Unlearning Club space and into day-to-day work. The structure of the club went beyond learning factual information; the discussions allowed participants to develop an awareness and understanding of how we are all interconnected within the broader context of oppressive structures or systems. Understanding relationality with systems of oppression involves acknowledging one’s role in these larger structures and recognizing the impact of systemic forces on individuals and communities. One participant noted *“it is affirming to hear how others feel and know I am not alone in my learning journey.”*

Trust played a central role, as individuals rely on each other to fulfill their commitments and obligations towards anti-racist initiatives. Fostering trust was in part due to the consistency of meeting with the same groups over the course of 17 months. This ‘closed cohort’ platform provided the time and space to build relational accountability. The participants acknowledged that both individual and collective contributions were needed for this work to be successful. Colleagues not only strengthened their intellectual understanding of systemic oppression and anti-Indigenous racism, but the growth of relational accountability created an imperative to unlearn and undo harmful practices and environments as a duty to their colleagues and as public servants. As hosts, we witnessed colleagues bringing their learning into day-to-day work of the OPHO, which is discussed in theme 4.

A significant reason we believe the club fostered these dynamics is our ‘Love and Care’ approach to this work, which allowed us to create a welcoming, compassionate atmosphere. These dynamics allowed individuals to express themselves in vulnerable ways, building trust and solidarity with their peers. Our love and care approach recognizes that sustainable change requires creating an empowering atmosphere where everyone feels they can bring their whole selves, which sometimes includes asking the hard questions, being uncomfortable, or prioritizing self-care. This was affirmed by participants, *“it’s good to be part of a group committed to this process of [un]learning. There is a feeling that we are there for each other in this hard heart work soaked in tragedy and tears.”*

Another aspect that fostered relational accountability was using a circle format for discussions. This was a deliberate tool to support Unlearning as it modeled an alternative to typical discussion formats in government meetings. Some were more accustomed to what we term “popcorn style” meetings, where putting hands up signals desire to speak, and often there is imbalance in airtime between participants. Using a circle to structure discussions was new for many Unlearning Club participants and created a different dynamic. Circle protocol fostered a sense of shared responsibility of the work required and created accountability & vulnerability within the group.

**Table:** 

Circle Protocol
The circle as a methodology reflects an Indigenous approach to teaching and learning, emphasizing interconnectedness and respect for all participants [41, 42].
**Configuration**: For an in-person circle setup, arrange chairs in a circle without barriers to ensure visibility and equality among participants. For online circles, use a video conferencing platform with a gallery view to mimic the circle arrangement.
**Discussion**: Whether in-person or online, the circle discussion process involves each participant taking turns to speak, usually in a predetermined sequence (often moving clockwise around the circle). This ensures that everyone has an equal opportunity to contribute without interruption, fostering a respectful and inclusive environment where all voices are heard and valued. The circle often goes around multiple times, allowing participants to build upon earlier thoughts or share new reflections as the discussion evolves.
**Sharing Personal Insights**: Participants are encouraged to contribute their own perspectives or experiences rather than directly responding to or debating points made by others. This approach helps to avoid confrontational dynamics and promotes a non-judgmental atmosphere.
**Option to ‘Pass’**: In the circle format, participants have the option to ‘pass’ if they do not wish to speak when their turn comes. This aspect of the circle is crucial in respecting individual comfort levels and readiness to share.
**Facilitator’s Role**: The facilitator guides the discussion, ensures that the circle’s principles are upheld, and helps to maintain a respectful and productive environment.
**Closure and Reflection**: Circles typically end with a process of closure and reflection, giving participants the opportunity to express how the dialogue has impacted them and to acknowledge the collective journey of the group.

3. **Sustained unlearning over time offered several unexpected benefits (individual journeys).**

The sustained commitment to unlearning over time yielded unexpected yet valuable benefits. The Unlearning Club’s 17 months of intentional engagement facilitated a transformative learning journey for participants. A key aspect of this sustained unlearning involved the *intentional* practice of systematically listening to Indigenous, Black, and People of Color voices each month, avoiding relegating these valuable teachings to a perpetual ‘to-read’ list. This 17-cycle commitment reflects a structured and disciplined approach to incorporating diverse perspectives into the learning journey. Notably, individuals actively brought the knowledge gained from the Unlearning Club into their daily work responsibilities, creating a continuous feedback loop between theoretical learning and practical application. As participants delved deeper into these issues, they found themselves unable to ‘unsee’ the ways in which white supremacy operates, leading to a lasting shift in perspective: *“recognition of my family and personal benefits from some of the racist policies*,*” “[I learnt] that racist policies are still all around us and we need to look for them and see them. There is no neutral: it is racist or anti-racist*.”. While this shift did not always immediately translate into action, participants recognized unlearning as an ongoing process rather than a one-time endeavor. The 17-month duration of the Unlearning Club underscores that dismantling white supremacy requires sustained effort, reflection, and discomfort—it is neither quick nor linear.

Another important outcome of the sustained unlearning was the shift in comfortableness with the materials over time. The initial Unlearning Club sessions saw strong reactions and realizations from participants, such as shock, guilt, discomfort. One participant noted they had *“lots of feelings arising – anger*, *frustration*, *sadness*, *feeling complicit*, *feeling defeated and not knowing how to be better*,*” “I am very disturbed by the fact that I am the age I am and am still learning about something that has been critically disruptive and harmful to generations of people.”* Sustained learning over 17 months allowed participants to sit with the difficult conversations and focus on the critical inner work that is essential in the ongoing journey towards genuine anti-racist action.

4. **Collective Journey: Unlearning Club helped to build a “we” or collective sense of attention and responsibility to the issue of Indigenous-specific anti-racism.**

The Unlearning Club was instrumental in cultivating a sense of collective consciousness and shared responsibility among its members. As relationships are forged, a deeper understanding of this work unfolds; people become united, driven by a desire to comprehend and alleviate another’s suffering, and committed to contributing to the solution. A strong community was built around the Unlearning Club, as one member shares: *“It is so valuable to have a cohort to do this work with*, *of like-minded people on the same journey. Maybe at different places in that journey but at least all pointed in the same direction…”* Too often, anti-racism work falls on the few Indigenous, Black, and People of Color in the workplace. We saw a clear shift in understanding during the Unlearning Club that this is work we can and will all be doing together. This idea goes hand-in-hand with one of our guiding principles of this work: no one should be doing this work alone. The connections made during the Unlearning Club emboldened people to face problems and challenges together, which in turn made people feel more comfortable to speak up and out against systemic injustice.

5. **Fighting Inertia: Participation in the Unlearning Club counters the narrative of resistance in the system.**

Contrary to the prevailing narrative that suggests there is resistance to anti-racism initiatives within systems, our experience demonstrates the opposite. Many individuals were not only eager but grateful for the chance to engage with cultural safety and anti-racism unlearning. When given the opportunity and backed by supportive leadership, the myth of systemic resistance crumbles. This is evidenced by the majority of staff within the OPHO participating in the Unlearning Club, which was completely voluntary. Additionally, the majority of participants readily and eagerly completed all the homework assigned each month. Leadership played a pivotal role in enabling this engagement. Participating in Unlearning Club was encouraged and modeled by senior leadership, and this included protecting time for staff to prepare for the monthly sessions. Being given permission to allocate work hours to complete the homework and participate in the sessions was a powerful demonstration of the office’s commitment to anti-racism. It was a clear message that integrating anti-racist practices is not peripheral but central to the OPHO’s mission, values, and operations. The encouragement from the leadership at the highest levels of the organization served as a strong endorsement for employees to weave these essential teachings into their daily work. Valuing and prioritizing such tasks effectively challenge the notion that there is not enough time and refutes the idea that anti-racism work is something to be done off the side of your desk. Enabling leadership, operational support (U&U team), and having a clear direction were all essential components to having successful voluntary buy-in, and negating resistance.

6. **Moving to Action: The framework helped us not get ‘stuck’ in unlearning but move into action.**

Using Camara Jones’ three tasks framework to structure our modules moved us from a state of passive learning to proactive engagement. By shifting our focus from the disparities, tragedies, and failings of our systems, and towards the underlying root causes and the intentional design of systemic oppression, we began to refine our analytical lens. Participants noted that past cultural safety teachings often stayed in the “factual” realm of learning and didn’t push participants to think critically about their individual role in racism and white supremacy. At first, participants often commented on problems of the system, but would not directly discuss their roles and places *within* the system. As we progressed, participants began to understand themselves within the structures of racism and white supremacy, and how their everyday actions contribute to the system, *“owning my whiteness (as much as I hate capitalizing it) so as not to deflect responsibility and culpability; sitting with the discomfort; honoring my journey*,*” “realizing I’ve never really had an idea clash with a Western mainstream approach.”* Within the participants’ rapid reflections, we asked them to share actions they took to undo systemic white supremacy or racism that month. Initially there were delays in people taking action as they slowly took in the material and the structure of this work. However, as we progressed, we began seeing detailed explanations of actions participants took in their day-to-day work. For example, one participant *“corrected an assumption that was made in e-mail by a colleague that First Nations communities are inherently vulnerable.”* It was also important that participants didn’t move directly into action without the skills of naming racism and understanding the root causes. Using the Camara Jones framework allowed us to unlearn the inherited white supremacy and racism in our sphere, and patiently and persistently work to undo it.

## Discussion

True anti-racist transformation demands sustained effort over time. The Unlearning Club served as a transformative action that advanced our collective responsibility to embed anti-racism and cultural safety practices into our day-to-day work. It emboldened OPHO’s ongoing efforts to earn and maintain the trust of Indigenous Peoples, as well as communities of Color in BC, through centering on unlearning and undoing systemic white supremacy and racism inherited from the settler colonial origins of our institutions.

The Unlearning Club responds to the responsibility we have to uphold Indigenous rights and reconcile with the original inhabitants of the territories where we live. We created an environment that encourages both the on-going practice of disrupting the dominance of settler approaches, and recognizing we have a legal obligation to do so. While literature has continued to emphasize the importance of this work being taken up by settlers and non-Indigenous people to avoid the decolonizing work falling solely on the shoulders of Indigenous Peoples, there is also a consensus in scholarly sources that tackling racism in health requires more than just awareness and superficial changes a seismic shift in the way health education and practice are approached is necessary [43–45]. 

There is a constellation of anti-racism education here in BC and across Turtle Island [46], we believe that a variety of quality unlearning and learning initiatives is necessary as evidence demonstrates that a single engagement on the topic of Indigenous-specific anti-racism and/or cultural safety is insufficient for system transformation [45]. Unlearning Club’s distinctive focus on self (relationship to settler colonialism), and learning together sets it apart from traditional training opportunities that are often anonymous, focused on other (cultural competency), occur outside of the day-to-day workplace, and prioritize individual over collective learning. We have seen that fostering accountability through ongoing dialogue and sustained, deeply personal engagement is needed to dismantle system-level drivers of Indigenous-specific racism. The integration of Dr. Camara Jones’ and Jody Wilson-Raybould’s frameworks further distinguishes the Unlearning Club by structuring unlearning through a cyclical process of learning, understanding, and acting. Additionally, the Unlearning Club’s adaptability has allowed it to be taken up in other contexts, such as within student-led groups at UBC, demonstrating its scalability and relevance beyond government offices. We call on institutions to take a central role in anti-racism work by adopting models like the Unlearning Club to assist in the crucial process of unlearning ingrained biases and fostering a commitment to *continuous* growth of being anti-racist.

Witnessing all participants learn, grow, and challenge the status quo together was a beautiful and hopeful sight for our health system’s future. It shows that people are not inherently racist, racism is learned and socialized through our colonial systems and can be unlearned and unpacked through understanding the truths of settler colonialism and white supremacy. For Indigenous colleagues in the office, the knowledge that their peers were actively engaged in this process fostered a sense of cultural safety, reinforcing an environment of mutual respect and understanding.

A key takeaway from this experience is the necessity for all institutions to transition away from a simple acknowledgment of racism and move towards action by allocating the necessary time, financial resources, and leadership support [47]. True commitment is demonstrated through actionable steps: funding initiatives that dismantle systemic barriers, dedicating time for employees to engage in anti-racism training during work hours, and having leadership not only endorse the work but also participate in these efforts. Enabling leadership signals an organization’s dedication to anti-racism while also empowering individuals to contribute meaningfully to these endeavors, ensuring that the move towards equity and inclusivity is both deliberate and sustained.

## Conclusion

The Unlearning Club has emerged as a pivotal strategy for fostering environments of cultural safety, anti-racism, and trustworthiness within our public health office. The key reflections underscore the value of sustained engagement with anti-racist education, demonstrating that with supportive leadership and an inclusive learning structure, resistance to anti-racism initiatives can be significantly reduced. Participants’ narratives reveal a transition from passive acknowledgement to active confrontation of systemic inequities, aided by frameworks such as Camara Jones & Jody Wilson Raybould.

We need all public health systems to implement sustained, structured education like the Unlearning Club to ensure anti-racism work is an institutional responsibility rather than an individual choice. It is not simply about understanding history, but involves a deeper, systemic overhaul that begins with education and evolves into a lifelong commitment to equity and justice. Commensurate with current U&U team capacity, we have launched a Round 2 Unlearning Club with a new curriculum tailored for the OPHO leadership team. Additionally, plans are in place to launch new cohorts utilizing the Round 1 curriculum, ensuring sustained learning opportunities. The Unlearning Club model is now being adopted in various settings across BC, including government ministries, regional health authorities, professional regulatory bodies, academic institutions, and beyond. This growing adoption highlights the scalability and impact in fostering anti-racism work. Given that this type of sustained education is largely absent in many public health settings, broader adoption of similar models is necessary.

As this journey continues, the hope is that the insights and reflections shared here will inspire and inform similar initiatives, fostering a wider movement towards a more culturally safe, equitable, and trustworthy public health landscape. The path is long, and the work is complex, but the Unlearning Club provides a testament to the profound impact that committed, collective action can have on our progress towards anti-racism.

## Limitations

We acknowledge that while the OPHO Unlearning Club focused on Indigenous-specific anti-racism, it is important that there is space to unlearn and undo other systems of oppression (e.g., ableism, homophobia and transphobia, classism) that permeate our institutions and policies. Our approach to unlearning and undoing must be intersectional to effectively solve the issues facing our public health system. Other limitations include voluntary participation in the Unlearning Club, which may have resulted in self-selection bias, with individuals already engaged in anti-racism work being more likely to participate. Further, while reflections captured meaningful shifts in perspectives, this study does not assess the long-term impact of the Unlearning Club on institutional policies or sustained behavioral change. Future research must assess the Unlearning Club’s effectiveness in fostering long-term shifts in policy and practice, as well as its role in strengthening anti-racism muscles.

## Data Availability

The participants of this study did not give written consent for their data to be shared publicly, so due to the sensitive nature of the research supporting data is not available. Please reach out to Dr. Kate Jongbloed should any questions arise kate.jongbloed@bccdc.ca.
